# Fear-of-intimacy-mediated zinc transport controls fat body cell dissociation through modulating Mmp activity in *Drosophila*

**DOI:** 10.1038/s41419-021-04147-z

**Published:** 2021-09-25

**Authors:** Tian Wei, Xiaowen Ji, Qunhui Yu, Guangying Li, Lei Wu, Yan Gao, Guiran Xiao

**Affiliations:** School of Food and Biological Engineering, Hefei University of Technology, Hefei, Anhui China

**Keywords:** Proteases, Metals, Cell adhesion, Biological metamorphosis, Homeostasis

## Abstract

Matrix metalloproteinases (Mmps) are pivotal extracellular proteinases that have been implicated in tumour invasion and metastasis. *Drosophila* fat body is important for energy storage and utilization, as well as biosynthetic and metabolic activities. The fat body undergoes remodelling during metamorphosis which is characterized by the dissociation of the fat body into individual cells. Mmps play important roles in the regulation of fat body cell dissociation. Here we show that a zinc transporter fear-of-intimacy (foi) is necessary for the cell dissociation of fat body in *Drosophila*. The progression of fat body cell dissociation was delayed by fat body-specific *foi* knockdown while it was accelerated by *foi* overexpression (OE). In essence, these phenotypes are closely associated with intracellular zinc homeostasis, which can be modulated by dietary zinc intervention or genetic modulation of other zinc transporters. Further study indicated that Mmp1 and Mmp2 levels could be transcriptionally regulated by zinc in vivo. Consistently, the retarded fat body cell dissociation caused by *Mmp1* or *Mmp2* RNAi could be regulated by modulating the expression of *foi*. Further, by using *Drosophila* models of malignant tumour Raf^GOF^scrib^−/−^ and Ras^V12^lgl^−/−^, we showed that the tumour growth, invasion and migration could be markedly inhibited by *foi* knockdown. These findings demonstrate a close connection between zinc levels and cell dissociation in vivo, and also suggest that manipulation of zinc levels may provide a novel therapeutic strategy for cancer.

## Introduction

Mammalian matrix metalloproteinases (Mmps) participated in the degradation of various proteins in the extracellular matrix (ECM) [1]. Mmps have been implicated in the migration of normal and malignant cells through the basement membrane (BM), a specialized form of ECM that subtends all epithelial cells [2]. Emerging evidence indicated aberrant Mmp expression has been associated with multiple stages of cancer progression including tumour invasion and metastasis [3]. However, most clinical trials of Mmp inhibitors have failed, so alternative drug candidates and therapeutic targets are urgently required [4, 5].

*Drosophila melanogaster* has gained appreciation as a useful model for studying genetics, developmental biology and human diseases [1, 6]. *Drosophila* fat body is a tissue that is analogous to vertebrate liver and adipose tissue, involving many important roles in metabolic activities [7–9]. *Drosophila* fat body undergoes remodelling during early stages of metamorphosis, and the fat body is gradually remodelled from a single-cell layer of attached polygonal cells into free floating as single cells or as small clusters [10]. Generally, the integrity of tissue or organ is maintained with BM in animals [11]. The destruction of cell–cell and cell–BM adhesion in fat body is a crucial step for the progression of cell dissociation [12]. There are two Mmps (Mmp1 and Mmp2) in *Drosophila* [13–15]. Previous work showed that Mmp1 and Mmp2 are required for fat body cell dissociation in *Drosophila* [16]. Mmp1 cleaves cell–cell junctions mediated by DE-cadherin, while Mmp2 degrades BM components maintained by integrin βPs [16]. The remodelling of fat body which involves Mmps is widely found in insects during metamorphosis, but the regulation of this process remains largely unknown.

Zinc is an essential trace element for all organisms [17, 18]. The SLC39 (Zrt/IRT-like proteins, ZIPs) family members regulate zinc influx from the extracellular medium or vesicular organelles into the cytoplasm, whereas the SLC30 (Zn-transporters, ZnTs) family members transport zinc in opposite orientations [19, 20]. Mmps could be activated by the expression of zinc transporters including ZIP5, ZIP6, ZIP7, ZIP8 and ZIP10 [21]. However, the regulatory mechanism of zinc on Mmps in vivo still remains unclear.

There are at least ten putative ZIP family members in *Drosophila* [19, 20, 22, 23]. Among them, fear-of-intimacy (foi)/CG6817 shares the highest overall homology with human ZIP6 and ZIP10 [20, 24, 25]. In both yeast and mammalian cell assays in vitro, foi was dissected as a zinc transporter localized to the cell plasma membrane [20, 25]. Foi has been found to be involved in several important physiological processes, such as gonad and trachea morphogenesis, myogenesis and glial cell migration in *Drosophila* [24, 26, 27].

In this study, we demonstrated that foi maintains zinc homeostasis in the fat body of *Drosophila*. Alteration in the expression of *foi* in the fat body, or modulation of dietary zinc availability, significantly modified the progression of fat body cell dissociation. Further study indicated that the altered transcription of Mmps is responsible for the modulation of zinc on fat body cell dissociation. In addition, using the *Drosophila* malignant tumour models, Raf^GOF^scrib^−/−^ and Ras^V12^lgl^−/−^, we found that the tumour growth, invasion and migration could be remarkably restrained by *foi* RNAi.

## Materials and methods

### *Drosophila* strains and culture media

Unless otherwise noted, flies were normally reared on standard cornmeal media at 25 °C. The following stocks were used: (1) *w1118* (V#60000), (2) *UAS-foi* RNAi (V#10102), (3) *UAS-Mmp1* RNAi (V#101505), (4) *UAS-Mmp2* RNAi (V#107888) were obtained from the Vienna *Drosophila* RNAi Center. (5) *Lsp2-Gal4* (B#6357) and (6) *Cg-Gal4* (B#7011) were obtained from the Bloomington *Drosophila* Stock Center. (7) *UAS-foi* OE [24] was a kind gift from Dr. Mark Van Doren. (8) *UAS-dZnT1* OE [28] was a kind gift from Dr. Bing Zhou. (9) Viking-GFP, (10) y,w,ey-flp; tub-Gal80 FRT40A; Act-y^+^-Gal4 UAS-GFP, (11) w;lgl^4^ FRT40A UAS-Ras^V12^/Cyo;Sb/TM6B, (12) y,w,ey-flp; Act-y^+^-Gal4 UAS-GFP; FRT82B tub-Gal80 and (13) w; Adv/Cyo; UAS-Raf^GOF^ FRT82B scrib^1^/TM6B were generously provided by Dr. Jose C. PASTOR-PAREJA.

The concentrations of supplemented metals or metal chelators used were as follows: 25 μM N, N, N′, N′-tetrakis (2-pyridylmethyl) ethylenediamine (TPEN) (Sigma, Cat#P4413), 2 mM ZnCl_2_ (Sigma, Cat#746355) and 2 μM Zinpyr-1 (Santa Cruz Biotechnology, sc-213182).

### Eclosion rate assay

*Cg-Gal4* homozygous flies were crossed to transgenic flies. After 3 days mating, the parents were transferred to juice-agar plates to lay eggs for 24 h. Newly hatched first-instar larvae were transferred to normal food. The density was controlled to 70 larvae/vial. The total number of emerging adults of each genotype was counted. Six parallel group tests were conducted for each genotype, and the experiments were repeated at least three times.

### Alkaline phosphatase (ALP) activity assay

Samples (fat bodies of 15 larvae) were lysed in ALP lysis buffer (10 mM Tris-HCl, 0.5 mM MgCl_2_ and 0.1% Triton X-100, pH 7.4), then 1~2 μg total protein was incubated in 1.0 M diethanolamine, 0.5 mM MgCl_2_ and 150 mM p-nitrophenyl phosphate (pNPP). The absorbance at 405 nm was measured after incubation for 30 min at 25 °C.

### Quantitative measurements of fat body cell dissociation

The fat body was dissected out from each animal at different developmental stages under Nikon camera. ImageJ software was employed to calculate the total area of fat-body tissues of each genotype and the area of its non-dissociated fat-body tissues (area non-dissociated). The degree of fat body cell dissociation was calculated using the following formula: Dissociation (%) = 100 % × (total area − non-dissociated area)/total area [16]. Dissociation (%) was compared among different genotypes at the indicated developmental stage. For analysing fat body cell dissociation of each genotype at one developmental stage, 3–10 animals were used for each independent replication and three independent replications were carried out.

### Quantitative measurements of gap area in the fat body

ImageJ software was employed to calculate the total area of fat-body tissues of each genotype and the area of gaps in fat-body tissues. The ratio of gap area in the fat bodies was calculated using the following formula: Gap area (%) = 100% × gap area/area total. Gap area (%) was compared among different genotypes at 7 h after puparium formation (APF).

### RNA isolation, semiquantitative RT-PCR and quantitative real-time PCR

Total RNA was extracted with Trizol reagent (Invitrogen, Carlsbad, CA, USA). cDNA was reversely transcribed from 2 μg total RNA with TransScript Reverse Transcriptase (TransGen Biotech Co, Beijing, China). Semiquantitative RT-PCR was performed using gene-specific primers to amplify partial regions of the gene foi. RNA isolation and reverse transcription were performed independently for three times.

qPCR was carried out in a 20 μl reaction volume containing 10 μl of 2× TransStart Green qPCR SuperMix (TransGen Biotech Co., Beijing, China), 1 μl of first-strand cDNA template (as prepared above) and 0.4 mM of each primer. The Real-Time PCR Detection System (Roche, Switzerland) was used according to the manufacturer’s instructions. The ribosomal protein 49 gene (rp49) was used as the control. RNA isolation and reverse transcription were performed independently for three times.

The primers used for RT-PCR and qPCR were:

foi-F: 5′-TGATTCATTTCCACGCAGTTC-3′,

foi-R: 5′-ATCCATATTGCTACGACGACGAGA-3′;

Mmp1-F: 5′-AGGACTCCAAGGTAGACACAC-3′,

Mmp1-R: 5′-TTGCCGTTCTTGTAGGTGAACGC-3′;

Mmp2-F: 5′-AACGACGACCGCATGAAGGTG-3′,

Mmp2-R: 5′-GAAGTGGTTGATCCTTAGCTCCC-3′;

rp49-F: 5′- GCACCAAGCACTTCATCC-3′,

rp49-R: 5′-CGATCTCGCCGCAGTAAA-3′.

### Immunohistochemistry

The third-instar larvae were dissected in cold PBS, the dissected tissues were fixed in 4% formaldehyde in PBS for 10 min at room temperature, stained and mounted following standard procedures [29]. The following antibodies and dyes were used: rat anti-DE-cadherin (DCAD2, 1:100 Developmental Studies Hybridoma Bank, DSHB, USA), mouse anti-Mmp1 (1:100 DSHB) and mouse anti-integrin βPS (CF. 6G11, 1:100, DSHB). The antibody of Mmp2 was gifted from Dr. Sheng Li [16]. The fluorescein-conjugated secondary antibodies used were FITC-conjugated goat anti-mouse IgG (1:500 for Mmp1, 1:500 for integrin βPS), goat anti-rabbit IgG (1:500 for Mmp2) and Cy3-conjugated goat anti-rat IgG (1:500 for DE-cadherin). For DAPI staining, samples were incubated in 50 ng/ml DAPI for 12 min. Slices were mounted with 50% glycerol/PBS. Confocal images were taken with a Zeiss LSM710 Meta confocal microscope.

For Zinpyr-1 staining, the dissected tissues were fixed in 4% formaldehyde in PBS for 10 min at room temperature, 2 μM Zinpyr-1 stained for 60 min at room temperature, washed thrice with PBS and mounted with 50% glycerol/PBS. The fluorescence signal was examined by Nikon Ti2 fluorescence microscope.

### Assessment of total Mmp enzyme activity by gelatin zymography

Gelatin zymography was employed to determine the total Mmp enzyme activity of fat body samples. Unless otherwise noted, proteins were made from 50 fat bodies. Briefly, the dissected fat bodies were washed with cold PBS for three times, and extracted with PBST containing protease inhibitors. Protein concentrations were measured by the BCA Protein Assay Kit (Cat#23227, Thermo Scientific). Proteins were loaded onto the gel with equal amounts of protein per lane. Samples were run on SDS-PAGE (10% gel containing 0.1% gelatin; Cat#10010328, Sinopharm Chemical Reagent Co., Ltd.). After electrophoresis, gels were incubated with Renaturing Buffer (prepare 200 ml of 2.5% v/v Triton X-100 in dH_2_O), then stained with Coomassie blue (prepare 1 l of 0.5% Coomassie blue R-250, 5% methanol and 10% acetic acid in dH_2_O) [30]. All the experiments were repeated at least three times. ImageJ software was employed to quantify the Mmp activity by calculating the hydrolysis area in the lane. All data were normalized to the level in controls.

### Fluorescence microscopy

The whole bodies of tumour larvae were observed with a Nikon ECLIPES Ti2-U microscope (Nikon, Tokyo, Japan) at 12 days after egg laying (AEL). The morphology analysis was performed as described previously [31]. More than ten tumour larvae were recorded per genotype, and each experiment was repeated at least three times.

To further investigate tumour growth, the cephalic complexes, including eye imaginal discs, brain lobes and ventral nerve cord (VNC), were dissected in cold PBS within 20 min, fixed in 4% paraformaldehyde for 10 min, washed once with PBS and imaged immediately at 12 days AEL. ImageJ was employed to quantify the size and fluorescence intensity of tumours [31].

### Statistical analysis

Data were analysed by Student’s *t* test between groups, and one-way analysis of variance (ANOVA) was used for multiple groups. Statistical results were presented as mean ± SEM. Asterisks indicate critical levels of significance (^*^P < 0.05, ^**^P < 0.01 and ^***^P < 0.001).

## Results

### Fat body modulation of foi affects the zinc homeostasis and the development of *Drosophila melanogaster*

**Fig. 1:**
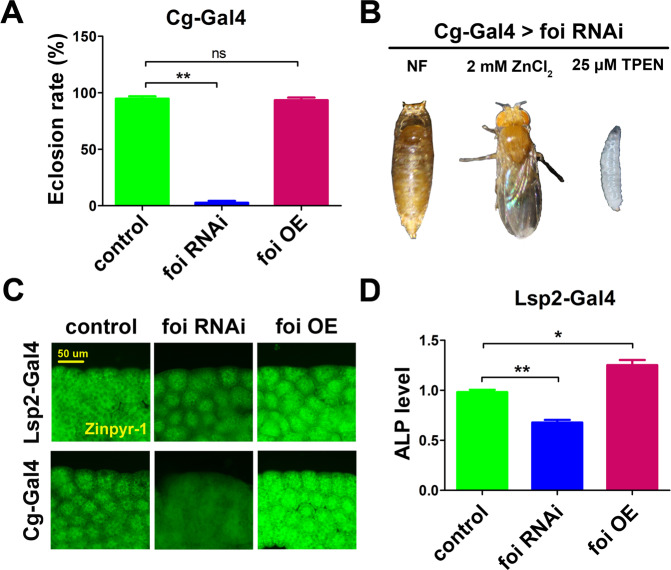
Foi maintains zinc homeostasis in the fat body and modulates the development of *Drosophila melanogaster*. **A** Fat body-specific *foi* knockdown (*Cg-Gal4* *>* *foi RNAi*) resulted in dramatically reduced eclosion rate in *Drosophila*. *n* = 70 larvae per vial, *n* = 6 vials per group. **B** The defects of RNA-interference *foi* (*Cg-Gal4* > *foi RNAi*) flies could be absolutely rescued by dietary zinc supplementation (2 mM ZnCl_2_) while aggravated by zinc chelator (25 mM TPEN). **C** The Zinpyr-1 staining revealed that fat body-specific *foi* OE resulted in zinc accumulation while *foi* RNAi decreased zinc particles in the fat body cells. **D** The activity of ALP was significantly reduced in *Lsp2-Gal4* > *foi RNAi* larvae while increased in *Lsp2-Gal4* > *foi OE* larvae. *w1118* flies driven by *Cg-Gal4* or *Lsp2-Gal4* serve as a blank control. All the experiments were conducted independently at least for 3 times. ns: not significant, ^*^P < 0.05, ^**^P < 0.01, ^***^P < 0.001, two-tailed Student’s *t* test.

*Cg-Gal4* is a driver specifically expressing the activator Gal4 in the fat body and haemocytes of flies [32, 33]. When *foi* was knocked down with *Cg-Gal4*, *Drosophila* displayed developmental arrest at the pupal stage (died prior to eclosion) (Fig. 1A). To distinguish the defects of *Cg-Gal4* > *foi RNAi* in the fat body or the haemocytes, *foi* was specifically knocked down in the haemocytes using a haemocyte-specific driver *He-Gal4* [33], no obvious defect was observed (data not shown). These data suggested that *foi* RNAi in the fat body resulted in developmental defects in *Drosophila*.

Interestingly, the eclosion rate of *Cg-Gal4* > *foi RNAi* was completely rescued by zinc supplementation in food from 3% to 100% (Fig. 1A, B), while all flies died at the second-instar larval stage when raised on zinc-deficient food (Fig. 1B). These data suggest that the expression of *foi* in fat body is important for development and the function of foi is closely related to zinc.

The role of foi in the fat body of *Drosophila* has not been investigated before. It has been reported that Zinpyr-1 is a zinc sensor that particularly amenable to intracellular work, in response to intracellular Zn^2+^ [34, 35]. As shown in Fig. 1C, zinc accumulation was observed in *foi* OE, while the zinc signal was attenuated in *foi* RNAi. *Cg-Gal4* is strongly expressed at all the stages of development [32]. In order to identify the function of foi in the fat body, *foi* RNAi was driven by the other fat body driver *Lsp2-Gal4*, which initiates expression in the fat body cells at the mid-instar transition midway through the third larval instar [36–38]. When the expression of *foi* was modulated by *Lsp2-Gal4*, the Zinpyr-1 staining results are consistent with *Cg-Gal4* (Fig. 1C). ALP activity is considered as a sensitive indicator of intracellular zinc level [39–41]. As shown in Fig. 1D, *foi* knockdown in the fat body led to a reduction of ALP activity, whereas *foi* OE resulted in elevated activity. Taken together, foi modulates zinc homeostasis in the fat body of *Drosophila*.

### The zinc homeostasis mediated by foi is required for fat body cell dissociation

It is known that the fat body remodelling is an essential developmental event which persists throughout pupal development [42]. We next investigated the influence of foi on the dissociation of the fat body into individual fat cells: they gradually become spherical and then physically detach from each other during the early pupal stage [16].

**Fig. 2:**
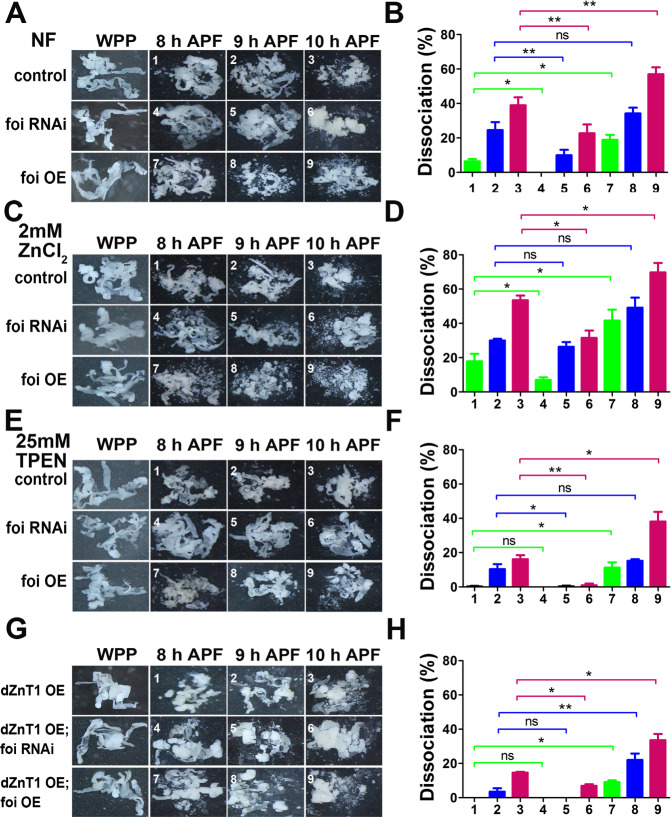
Cytosolic zinc affects the progression of the fat body cell dissociation. **A**, **B** The fat body cell dissociation was greatly delayed in *Lsp2-Gal4* > *foi RNAi* while accelerated in *Lsp2-Gal4* > *foi OE*. **C**, **D** Dietary zinc supplementation was able to rescue the retarded fat body cell dissociation induced by fat body-specific *foi* RNAi, while enhance the precocious the progression induced by *foi* OE. **E**, **F** Zinc chelator TPEN had the capability of rescuing the precocious fat body cell dissociation induced by *foi* OE, while aggravating the retarded fat body cell dissociation induced by *foi* RNAi. **G**, **H** *ZnT1* OE in the fat body dramatically rescued the precocious cell dissociation caused by fat body-specific *foi* OE, while enhanced the retarded cell dissociation induced by *foi* RNAi. All the experiments were conducted independently at least for 3 times. ns: not significant, ^*^P < 0.05, ^**^P < 0.01, ^***^P < 0.001, two-tailed Student’s *t* test.

In order to directly observe the morphological changes, we dissected the fat body of flies from the white prepupal stage (WPP) to 10 h APF and calculated the ratio of fat body cell dissociation. The fat bodies of flies were dissected out to monitor cell dissociation at 8, 9 and 10 h APF. As shown in Fig. 2A, under our experimental conditions, the fat body cells of wild-type flies intimately adhere to each other and form a single-cell layer of tissues until 8 h APF and appear with obvious cell dissociation at 10 APF. According to the results, the rate of cell dissociation was significantly reduced in *foi RNAi* (~23%) while being dramatically increased in *foi OE* (~57%), compared to control (~39%) at 10 h APF (Fig. 2B). These results reveal that foi manipulates the progression of the fat body cell dissociation.

Then we tested whether dietary zinc intervention could affect the modulation of foi on cell dissociation. The results showed that zinc supplement rescued the retarded cell dissociation caused by *foi* RNAi and enhanced the precocious dissociation caused by *foi* OE (Fig. 2C), showing a 9% and 13% increase in dissociation rate, respectively (Fig. 2D). By contrast, zinc depletion in food significantly inhibited cell dissociation of fat body at 10 h APF (Fig. 2E), with 22% and 19% decrease in *foi RNAi* and *foi OE* animals, respectively (Fig. 2F). In addition, compared with normal diet, zinc supplementation induced the fat body cell dissociation (from 39% to 54%), while zinc deficiency reduced this progression (from 39% to 18%) in wild-type animals at 10 h APF (Fig. 2A–F). This indicates that the regulation of foi on the progression of the fat body cell dissociation could be modulated by dietary intervention of zinc and further strengthens the notion that zinc is involved in the fat body cell dissociation.

As an alternative to examine the role of zinc homeostasis in the progression of the fat body cell dissociation, a well-established plasma membrane zinc transporter dZnT1/CG17723 (also named ZnT63C) [41] was selected to test the effect on fat body cell dissociation. dZnT1 functions in the opposite direction with foi [20, 28]. We observed that fat body-specific *dZnT1* OE vastly postponed the fat body cell dissociation (from 39% to 15%) and its dissociation rate was even lower than that of *foi* RNAi (~23%) at 10 h APF. Besides, the retarded fat body cell dissociation of *foi* RNAi could be aggravated by *dZnT1* OE (from 23% to 7% at 10 h APF); *dZnT1* OE was able to rescue the premature cell dissociation caused by *foi* OE (from 15% to 34% at 10 h APF) (Fig. 2G, H). This suggests that cytoplasmic zinc promotes the fat body cell dissociation.

Taken together, our results reveal that foi acts a pivotal part in the fat body cell dissociation; moreover, the function of foi on fat body cell dissociation is powerfully associated with cytoplasmic zinc levels.

### Foi is required for the structural stabilization of fat body cells

**Fig. 3:**
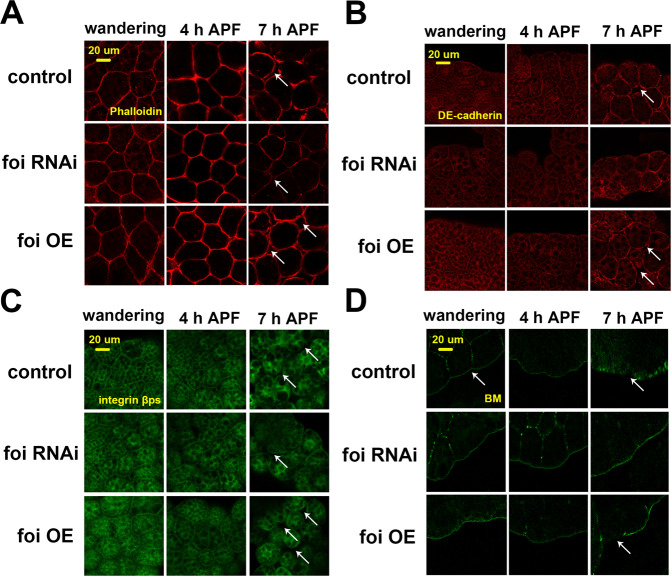
Foi is required for the structural stabilization of fat body cells. The fat body structure was investigated with four hallmarks at the wandering stage, 4 h APF and 7 h APF. **A** Phalloidin staining revealed the shape of the fat body cells. **B**, **C** Immunostaining of DE-cadherin (**B**) and integrin βPs (**C**) were used to monitor the cell–cell junctions and cell–basement membrane (BM) junctions, respectively. **D** Viking-GFP revealed the integrity of the BM. A nearly intact BM was observed in the fat body of *Lsp2-Gal4* > *foi RNAi* animals, while the BM was broken in *Lsp2-Gal4* > *foi OE* animals. *w1118* flies driven by *Lsp2-Gal4* serve as a blank control. The arrows in (**A**–**C**) indicated the gaps between cells, and the arrows in (**D**) showed the degraded BM.

Tissue remodelling has been reported to be involved in normal development, pathological wound healing, cancer invasion and metastasis [11, 16, 43]. Cell–cell and cell–BM junctions are disrupted during *Drosophila* fat body remodelling [16]. To reveal the cell shape, the phalloidin staining of F-actin, a key component of the cytoskeleton was determined here (Fig. 3A). The two important adhesion proteins DE-cadherin and integrin βPs were employed to monitor cell–cell and cell–BM junctions, respectively (Fig. 3B, C). With the help of these hallmarks, the critical time of fat body remodelling could be detected. We carefully dissected the fat body of *foi OE* animals at 8 APF and found that their fat bodies are easily disintegrated during immunostaining; therefore, we examined the four hallmarks of developmental profiles in the remodelling fat body at wandering stage, 4 h and 7 h APF. Various staining methods indicated that intercellular gaps were present in *foi OE* at 7 h APF, whereas cell-to-cell adhesion was relatively tight in *foi RNAi* (Fig. 3A–C). We quantified the gap area of the fat body cell dissociation at 7 h APF and found that the gap area was strongly increased by 6% in *foi OE* flies, while almost disappeared in *foi RNAi* flies, in comparison to wild-type flies (Fig. S1A). Interestingly, neither *foi* knockdown nor OE has any effect on the expression of both DE-cadherin and integrin βPs (Fig. S1B, C).

The intact BM is gradually destroyed until nearly disappeared at the late stage after puparium formation [16]. Viking-GFP is a functional GFP-trap fusion into the Collagen IV chain vkg, revealing the integrity of BM [16]. Robust GFP signal exhibited in *foi RNAi* files which indicated integrated BM, while the signal nearly disappeared in *foi OE* flies at 7 h APF (Fig. 3D). Furthermore, statistical analysis showed that fluorescence signal (Viking-GFP) was significantly reduced in wild-type larvae at 7 h APF, compared to the larvae at wandering stage or 4 h APF (Fig. S1D). The data were consistent with previous report that BM was gradually destroyed during development [16]. The signal in *foi* OE was weaker (reduced ~32%) than that in wild-type at 7 h APF. *foi* RNAi showed no significant difference in fluorescence signal at the three time points of development (Fig. S1D). Taken together, these results demonstrated that foi modulates the adhesion and structural stabilization between cells.

### Foi regulates the transcription of Mmps

**Fig. 4:**
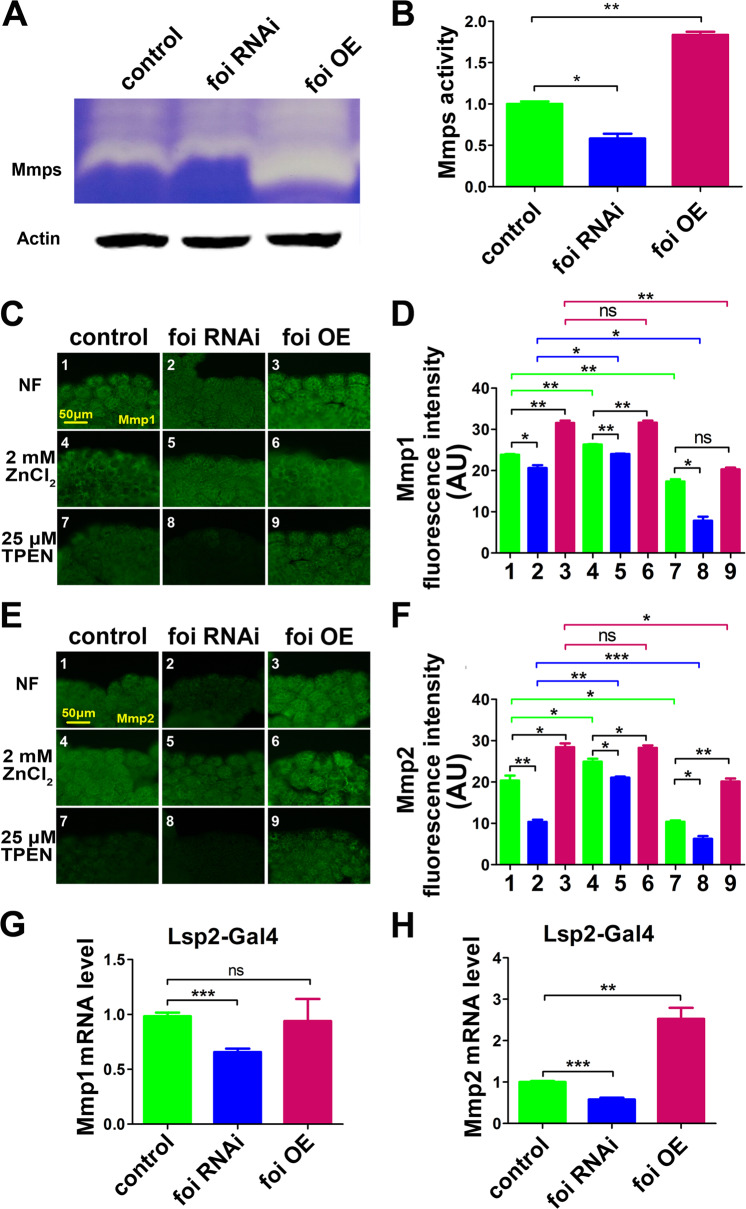
Zinc affects the enzyme activity, protein and mRNA levels of the two Mmps. **A** Gelatin zymogram revealed that total Mmp enzyme activity was considerably diminished in *Lsp2-Gal4* > *foi RNAi* larvae while increased in *Lsp2-Gal4* > *foi OE* larvae. **B** Quantitative measurement of (**A**). **C**–**F** Protein levels of both Mmp1 and Mmp2 in the fat body were reduced in *Lsp2-Gal4* > *foi RNAi* larvae while increased in *Lsp2-Gal4* > *foi OE* larvae. Dietary zinc supplementation was able to elevate the expression of Mmps, whereas zinc chelator TPEN can reduce their expression. **G**, **H** Both *Mmp1* and *Mmp2* mRNA levels were significantly down-regulated in *Lsp2-Gal4* > *foi RNAi* larvae, whereas a dramatically up-regulated *Mmp2* mRNA level could be detected in *Lsp2-Gal4* > *foi OE* larvae. *w1118* flies driven by *Lsp2-Gal4* serve as a blank control. All the experiments were conducted independently at least for 3 times. ns: not significant, ^*^P < 0.05, ^**^P < 0.01, ^***^P < 0.001, two-tailed Student’s *t* test.

To further identify the mechanism underlying foi’s effect on the progression of fat body cell dissociation, the activity of Mmps was examined (Fig. 4A, B). Gelatin zymography [30] indicated that the total Mmp enzyme activity of fat body was significantly reduced in *foi RNAi* larvae but increased in *foi OE* larvae (Fig. 4A, B).

Mmp activity is controlled by a complex array of mechanisms such as the level of gene expression, mRNA translation and protein modification [44]. Anti-Mmp1 and anti-Mmp2 could be used to detect the level of Mmp1 [16] and Mmp2 in vivo (Fig. S2). Immunostaining suggested that the expressions of Mmp1 and Mmp2 were decreased (14% and 49%, respectively) in *foi* RNAi while increased (32% and 40%, respectively) in *foi* OE (Fig. 4C–F). This result was also confirmed by western blot analysis (Fig. S3). What is more, the expression of Mmps could be elevated by zinc and inhibited by TPEN (Fig. 4C–F). Subsequently, q-PCR results indicated that the mRNA levels of Mmp1 and Mmp2 were significantly down-regulated in *foi RNAi* larvae (Fig. 4G, H). The *Mmp1* mRNA level showed slight up-regulation but without statistical significance (Fig. 4G); a dramatically up-regulated *Mmp2* mRNA level could be observed in *foi OE* larvae (Fig. 4H).

Accordingly, the gene expression profiles of *foi* and *Mmps* in the fat body of wild-type flies (*w1118*) were examined by RT-PCR analysis at different time points of development, including the wandering stage, WPP and 8 h APF (Fig. S4). We found that gene expressions of *foi*, *Mmp1* and *Mmp2* were gradually elevated during the development (Fig. S4A). Zinc levels were also elevated during the development (Fig. S4B). All the above data indicated that developmental changes of *foi* in the fat body result in elevated zinc levels in cells, which will induce the transcription of *Mmp1* and *Mmp2* during development. The induced expression and protein activities of Mmp1 and Mmp2 are responsible for the modulation of fat body cell dissociation.

### The fat body cell dissociation defects caused by *Mmp*s knockdown could be altered by foi level

**Fig. 5:**
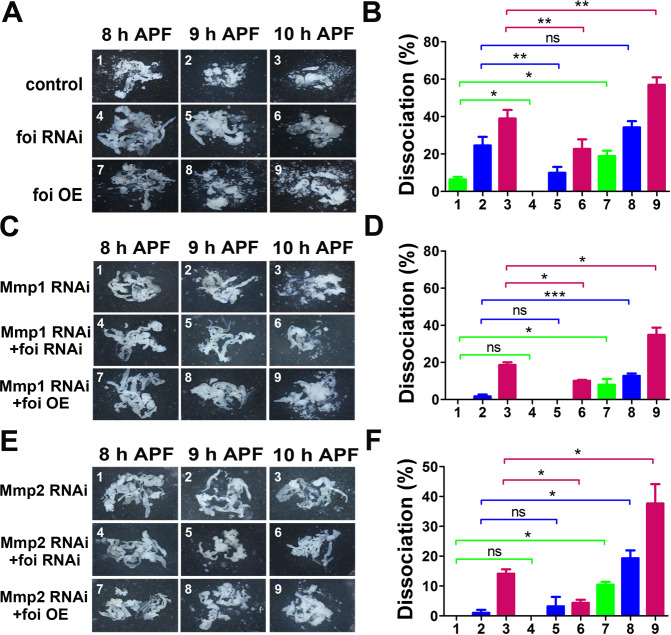
Retarded fat body cell dissociation caused by *Mmps* knockdown could be rescued by *foi* OE. **A**, **B** Fat body cell dissociation was significantly delayed in *Lsp2-Gal4* > *foi RNAi*, whereas a significant advance in fat body cell dissociation was also observed in *Lsp2-Gal4* > *foi OE*. **C**, **D** The retarded fat body cell dissociation caused by *Mmp1* RNAi could be enhanced by *foi RNAi* while rescued by *foi OE*. **E**, **F** *foi* RNAi aggravated the retarded fat body cell dissociation caused by *Mmp2* RNAi, while *foi* OE rescued it. All the experiments were conducted independently at least for 3 times. ns: not significant, ^*^P < 0.05, ^**^P < 0.01, ^***^P < 0.001, two-tailed Student’s *t* test.

Previous work has shown that the fat body cell dissociation was significantly delayed in *Mmp1 RNAi* and *Mmp2 RNAi* at the late stage of APF [16]. In order to further confirm the functional interaction between Mmps and foi, we used a combination of genetic approaches to investigate the effects of foi on the defects of *Mmps*’ RNAi in *Drosophila*. As revealed by Fig. 5A–F, the retarded cell dissociation in the fat body of *Mmp1* RNAi and *Mmp2 RNAi* was aggravated by *foi* RNAi (dissociation rate was from 19% to 10% and from 14% to 4%, respectively), and rescued by *foi* OE (dissociation rate was raised to 35% and 38%, respectively). All these data suggested that modulating zinc homeostasis in cytoplasm by altering the expression of foi could affect the defects of *Mmps* knockdown.

### The mechanism of foi regulates cell dissociation would be applied in modulating tumour invasion

**Fig. 6:**
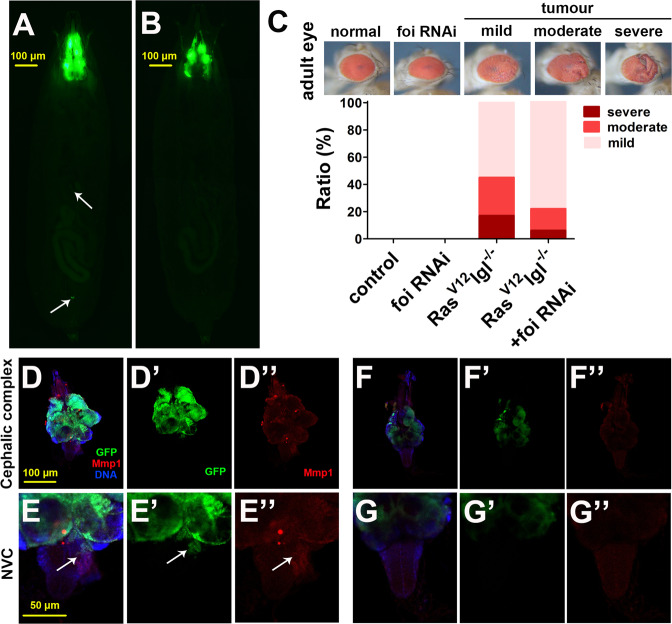
*Foi* knocked down in tumour cells repressed tumour growth and invasion. **A**, **B** Tumour growth and metastasis was observed in Raf^GOF^scrib^−/−^ at 12 days AEL (**A**), which can be dramatically suppressed by *foi* RNAi (**B**). **C** *Foi* knockdown inhibited tumour phenotypes of Ras^V12^lgl^−/−^ (*n* > 200 flies per group). When driven by eye-specific *ey-Gal4*, the *foi* RNAi did not cause any noticeable change in the morphology; *w1118* flies driven by *ey-Gal4* serve as a blank control. **D**, **E** Fluorescence micrographs of tumour cells and Mmp1 of eye-antennal disc and VNC were shown. Raf^GOF^scrib^−/−^ flies displayed overgrowth in the cephalic complex (GFP signal), invasive behaviour and massive Mmp1 activation (red signal, white arrowheads). Nuclei were stained with DAPI. **F**, **G** Silencing *foi* in the tumour clones strongly reduced the tumour growth, invasive behaviour and Mmp1 activation.

In *Drosophila* eye-antennal discs, cooperation between the classical oncoprotein Ras^V12^ or Raf^GOF^ and tumour suppressor lethal giant larvae (lgl) or scribbled (scrib) mutation gives rise to metastatic tumour model that exhibits many phenotypic characteristics observed in human cancers [45, 46]. It is reported that the massive Mmp1 activation is essential for BM degradation and epithelial to mesenchymal transition (EMT) progression in malignant Raf^GOF^scrib^−/−^ flies [47, 48]. We hence wondered whether the cellular zinc could regulate tumour growth and invasion in vivo. Tumour cells overgrew in the eye-antennal discs of the malignant model Raf^GOF^scrib^−/−^ flies and metastasized into several places (Fig. 6A). Amazingly, silencing *foi* in tumour clones led to a significant reduction in tumour growth and metastasis (Fig. 6B). As a confirmation, eye defect caused by another weaker tumour model Ras^V12^lgl^−/−^ was strongly rescued by *foi* knockdown (Fig. 6C). As shown in Fig. 6D, the tumour cells displayed overgrowth in the cephalic complex of Raf^GOF^scrib^−/−^ flies and invaded into the VNC at 12 days AEL (Fig. 6E). Silencing *foi* in the tumour clones strongly alleviated the tumour growth (the tumour size and fluorescence intensity of cephalic complex reduced by 42% and 38%, respectively, Figs. 6F and S5A, B) and invasive behaviour (Fig. 6G). The activated Mmp1 expression in Raf^GOF^scrib^−/−^ flies was significantly suppressed when *foi* was knocked down in the tumour cells (Fig. 6D–G). A statistical analysis of these data found that Mmp1 level of cephalic complex or ventral nerve cord (VNC) was remarkably reduced in *foi* RNAi tumour flies, showing 52% and 40% decrease, respectively, compared to Raf^GOF^scrib^−/−^ flies (Fig. S5C, D). Accordingly, the ALP activity in tumours with or without *foi* RNAi was detected, the results showed that zinc level was attenuated by 39% in *foi* RNAi, compared with Raf^GOF^scrib^−/−^ larvae (Fig. S6). These results suggest that reducing cytosolic zinc by inhibition of *foi* expression in tumour tissues could prevent tumour growth and invasion by reducing Mmp1 levels in *Drosophila*.

## Discussion

**Fig. 7:**
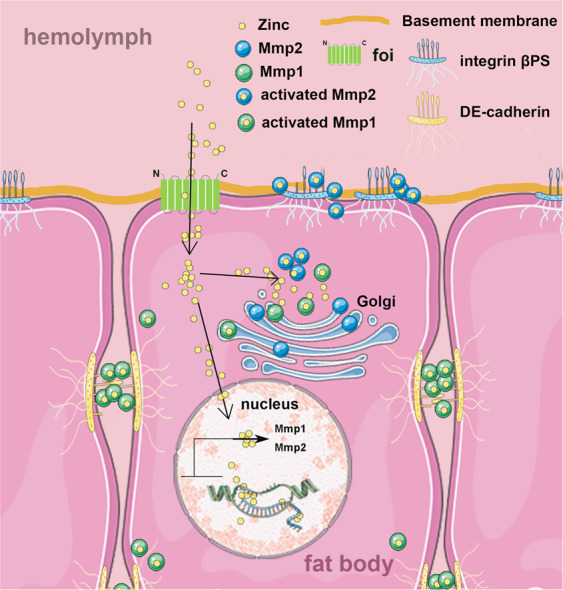
A model to explain the effect of foi on cell dissociation. In the fat body of *Drosophila melanogaster*, foi transports zinc into cytosol. In addition to direct activating Mmp activity, the increased zinc level in the cytosol also results in increased zinc in the nucleus which could induce the transcription of *Mmp1* and *Mmp2*. Mmps are involved in the progression of cell dissociation by promoting the degradation and hydrolysis of extracellular matrix components.

Cell dissociation is a process involved in many biological processes [24, 26] and some pathological processes, including tumour invasion and metastasis [49]. However, there are many details about the physiological importance or the regulation of this process that need to be clarified. The specific and efficient regulation of cell dissociation is absent till now. The insect fat body undergoes a dramatic remodelling process during metamorphosis in holometabolous insects [50]. Here we indicated *Drosophila* zinc transporter foi is necessary for the cell dissociation of fat body. Altered transcription of *Mmp1* and *Mmp2* in *foi RNAi* and *foi OE* is responsible for the regulation of fat body cell dissociation. These molecular and morphology phenotypes of *foi RNAi* and *foi OE* could be regulated by dietary zinc intervention. All the data strengthen the notion that zinc facilitates the cell dissociation of fat body in *Drosophila* by controlling *Mmp* expression. A model to explain the foi’s effect on fat body cell dissociation is shown in Fig. 7.

Zinc is an essential trace element in cells where it participates in a variety of fundamental biological processes [20, 51]. Mmps are expressed as inactive pro-enzymes, where the catalytic domain is shielded by a pro-domain that interacts with the zinc ion in the catalytic centre via a cysteine residue [52, 53]. The Mmps have a common zinc-binding motif (HEXXHXXGXXH) in their active site, and their domains contain one catalytic zinc and one structural zinc [54]. The catalytic site becomes accessible to substrates through removal of the pro-peptide [52, 53]. The endopeptidase activity is mediated by a zinc ion coordinated between three histidine residues that are present in the catalytic centre, and zinc ions directly participate in the bond-breaking step in the catalytic domain of Mmps [52, 53]. In vitro evidence indicated that Mmp activity could be activated by zinc while suppressed by removal or chelation of zinc from its active site [55–57]. Besides, Yoo and his colleagues [58] reported that the Mmp9 activity in the brain of ZnT3-null male mice was increased because of the increased cytosolic free zinc levels.

Zinc is important for its role not only in enzyme catalysis but also in regulation on gene transcription [59]. It is reported that approximately 40% of putative zinc-binding proteins are transcription factors, which are essential for regulating gene expression [60, 61]. These enable specific gene regulatory processes to be activated [60]. Here we showed that the mRNA and protein level of Mmp1 and Mmp2 could be modulated by the cellular zinc levels mediated by zinc transporter foi. Our findings expand our knowledge of Mmp activity in development and show that cytosolic zinc level promotes cell dissociation of fat body cells by governing Mmp activity. According to our findings, zinc may regulate the activity of Mmps through two aspects: on the one hand, zinc directly binds an extended zinc-binding motif of Mmps to modulate their activities; on the other hand, zinc modulates Mmps’ transcription to regulate their protein levels. But how the regulation of zinc modulates the transcription of Mmps awaits further investigation.

Interestingly, foi is homolog to mammalian ZIP6 and ZIP10 [20, 62]. It has been reported that the zinc transporters ZIP6 and ZIP10 are highly expressed in several breast cancers and associate with cancer invasion and metastasis [63–65]. ZIP6 reduces the expression of E-cadherin in mammalian breast cancer cells and forms a heteromer with ZIP10, contributing to cell migration process [63, 66, 67]. EMT is a process to investigate cancer cell migration, invasion and metastatic dissemination [68]. During the EMT, non-motile epithelial cells are dispersed into individual motile and invasive cells by dissolving the cell–cell and cell–BM junctions which is involved in Mmps [66, 68, 69]. Besides, foi is involved in regulating cell migration in some tissues of *Drosophila* [26, 70]. So we propose that foi may participate in the development and progression of cancer. With the help of the *Drosophila* malignant tumour model, Raf^GOF^scrib^−/−^ and Ras^V12^lgl^−/−^, we found that the tumour overgrowth, invasion and distant metastasis could be inhibited by *foi* knockdown in tumour clones. So we provided in vivo genetic evidence that manipulating zinc transporters can greatly modulate cancer progress. Moreover, we figure out the underlying mechanism is that the intracellular zinc regulates the expression of Mmps in vivo and then modulates their activities.

## Supplementary information

Supplemental material.

## Data Availability

The data that support the findings of this study are available from the corresponding author upon reasonable request.
