# Supplementary material for: Fear-of-intimacy-mediated zinc transport controls fat body cell dissociation through modulating Mmp activity in Drosophila
Source: Cell Death Dis. 2021 Sep 25;12(10):874. doi: 10.1038/s41419-021-04147-z (PMC8464599; doi:10.1038/s41419-021-04147-z)
Supplement: Supplementary file 1 — Supplemental material. [file 41419_2021_4147_MOESM1_ESM.doc]

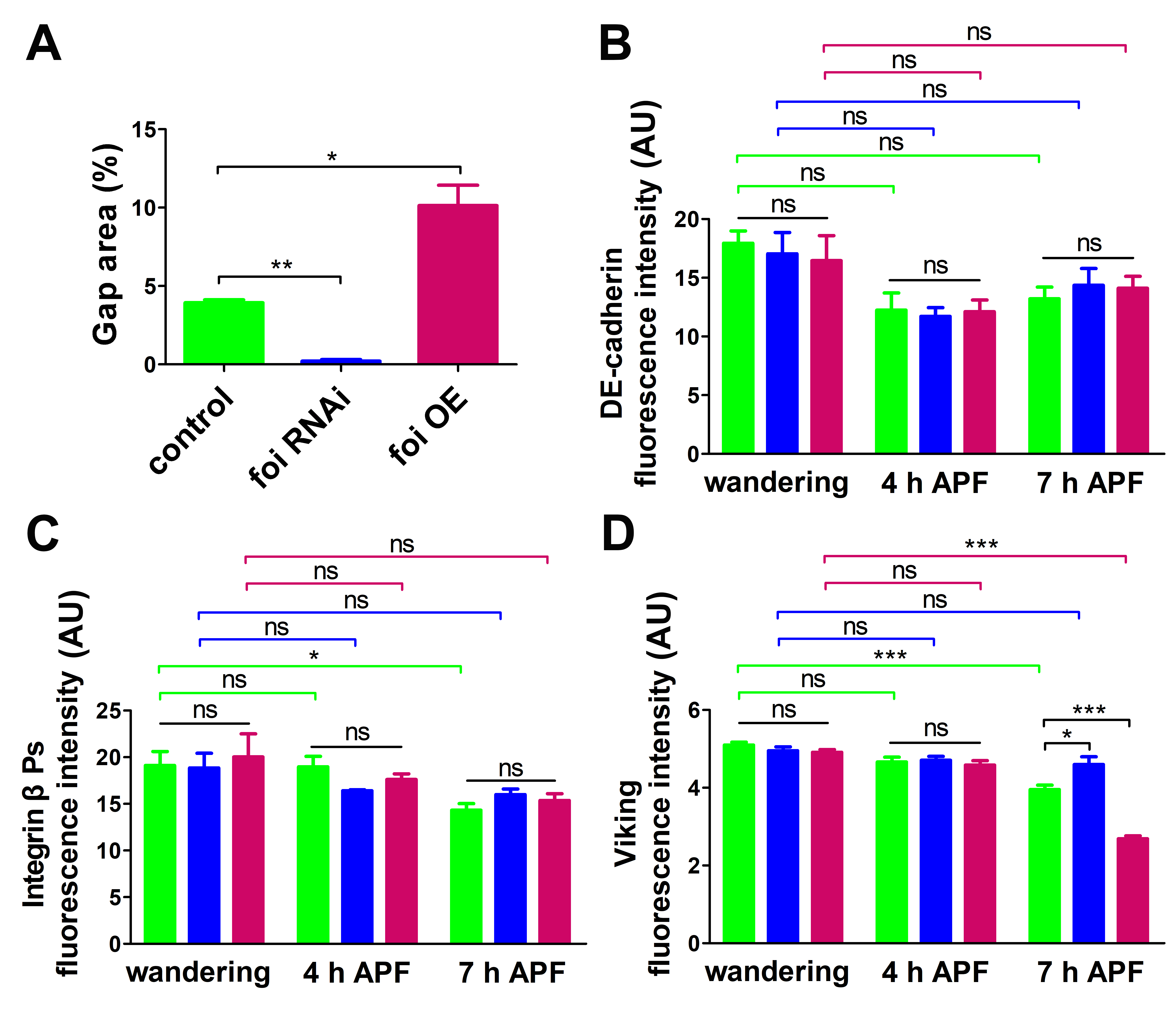


**Figure S1. Quantitative measurements of the gap area and related hallmarks in fat body.** (A) The gap area was significantly reduced (~4 %) in *Lsp2-Gal4>foi RNAi* larvae while being dramatically enhanced (~6 %) in *Lsp2-Gal4>foi OE* larvae, compared to control larvae at 7 h APF. (B-D) Quantification of DE-Cadherin, integrin βPs and Viking expression in the fat body at different time points of development, including the wandering stage, 4 h APF and 7 h APF. *w1118* flies driven by *Lsp2*-Gal4 serve as a blank control. ns: no significant, * *P* < 0.05, ** *P* <0.01, *** *P* <0.001, two-tailed Student’s t test.


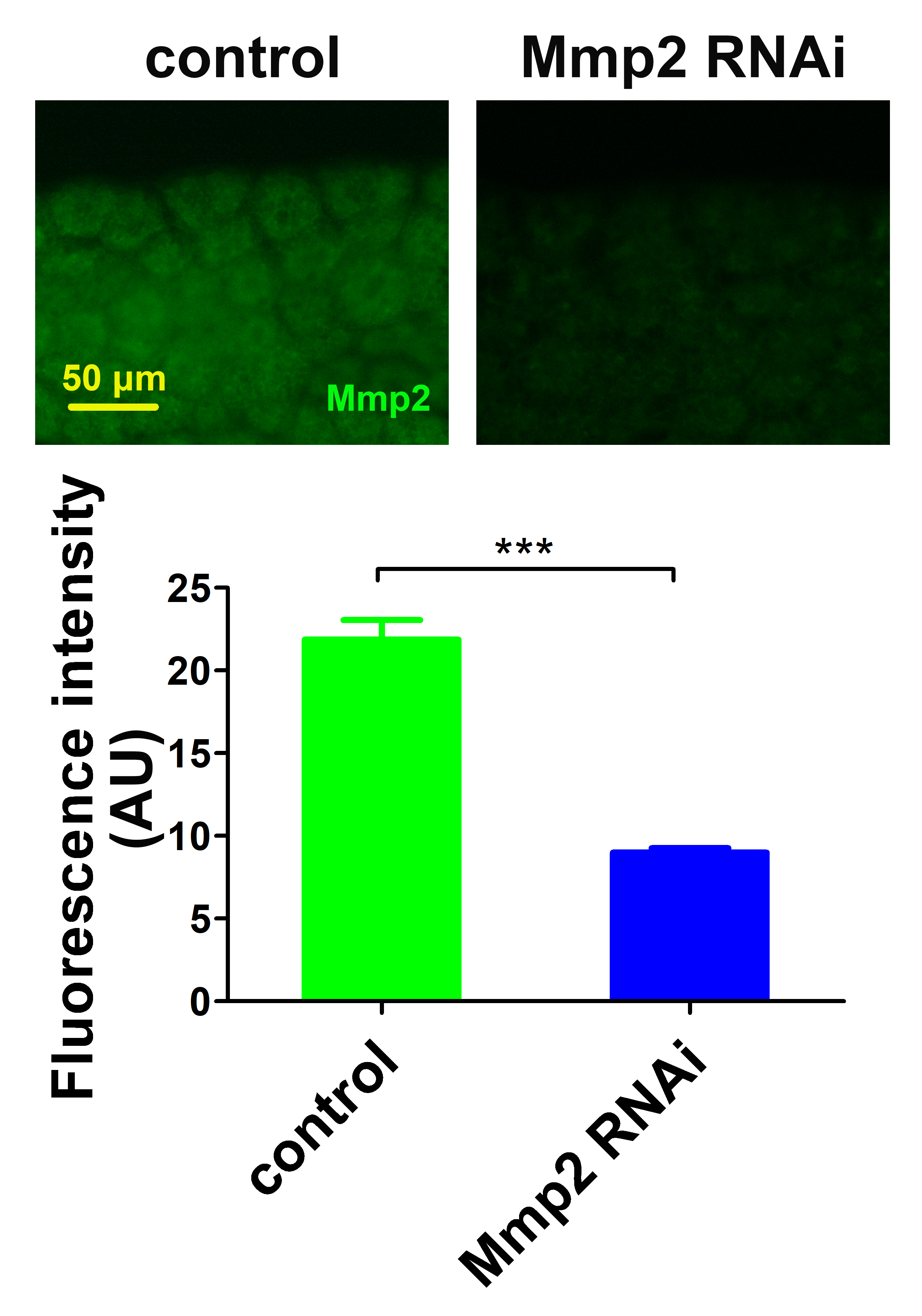


**Figure S2. Mmp2 anti-body could be used to examine the Mmp2 protein expression of fat body.** When Mmp2 was knocked down in the fat body, the fluorescence intensity dropped sharply, with an approximate 2.5-fold reduction in signal contrast (21.8 ± 2.7 vs 9.0 ± 0.6, p < 0.001). *w1118* flies driven by *Lsp2*-GAL4 serve as a blank control.


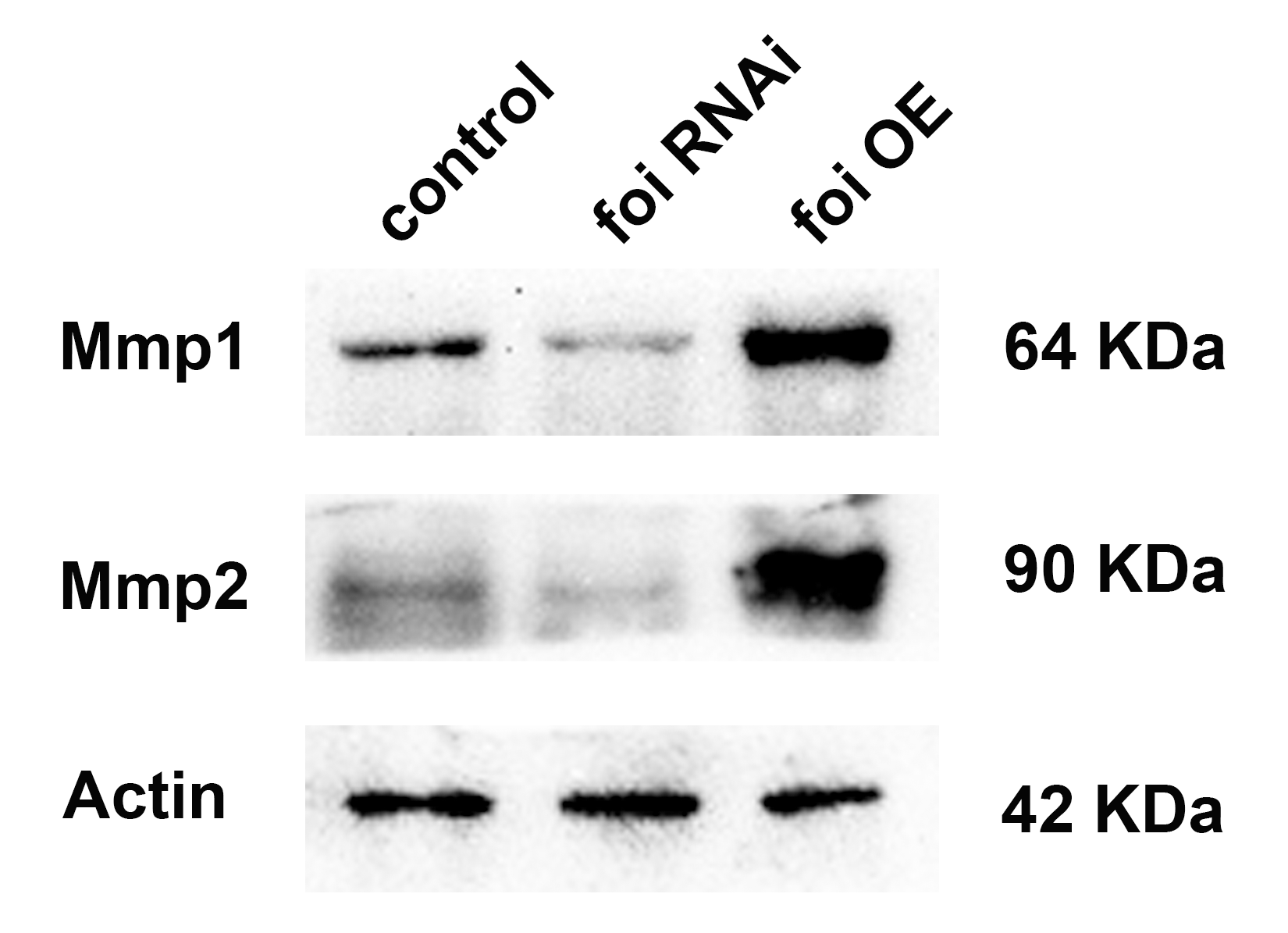


**Figure S3. *foi* RNAi resulted in reduction of both Mmp1 and Mmp2 protein expression, while *foi* OE elevated their expression.** *w1118* flies driven by *Lsp2*-GAL4 serve as a blank control. Actin was used as the loading control. n = 15 fat bodies of flies per group.


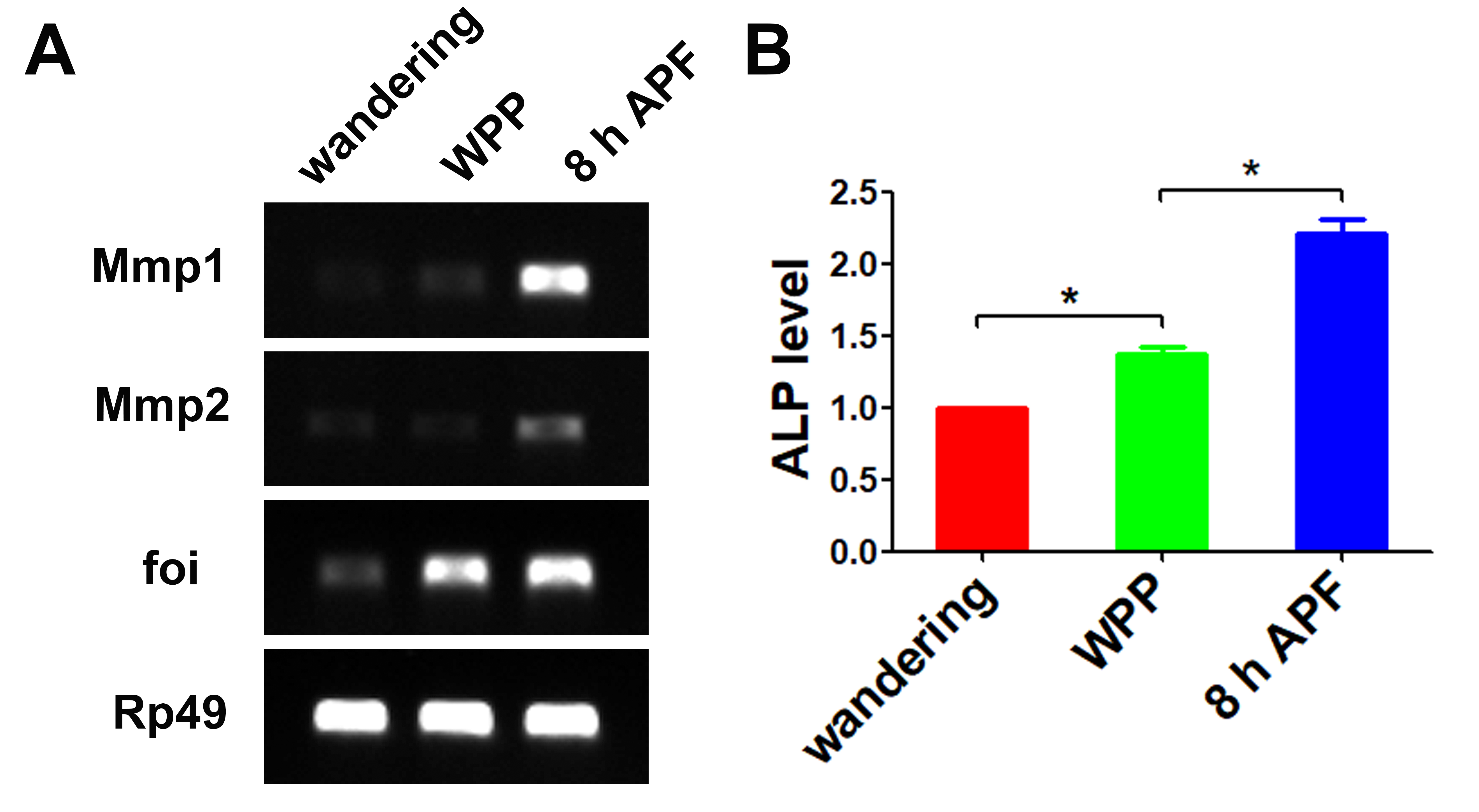


**Figure S4. Expression profiles of related genes and developmental profiles of ALP activity in the fat body.** All the experiments were conducted at least for 3 independent times. ns: no significant, * *P*<0.05.


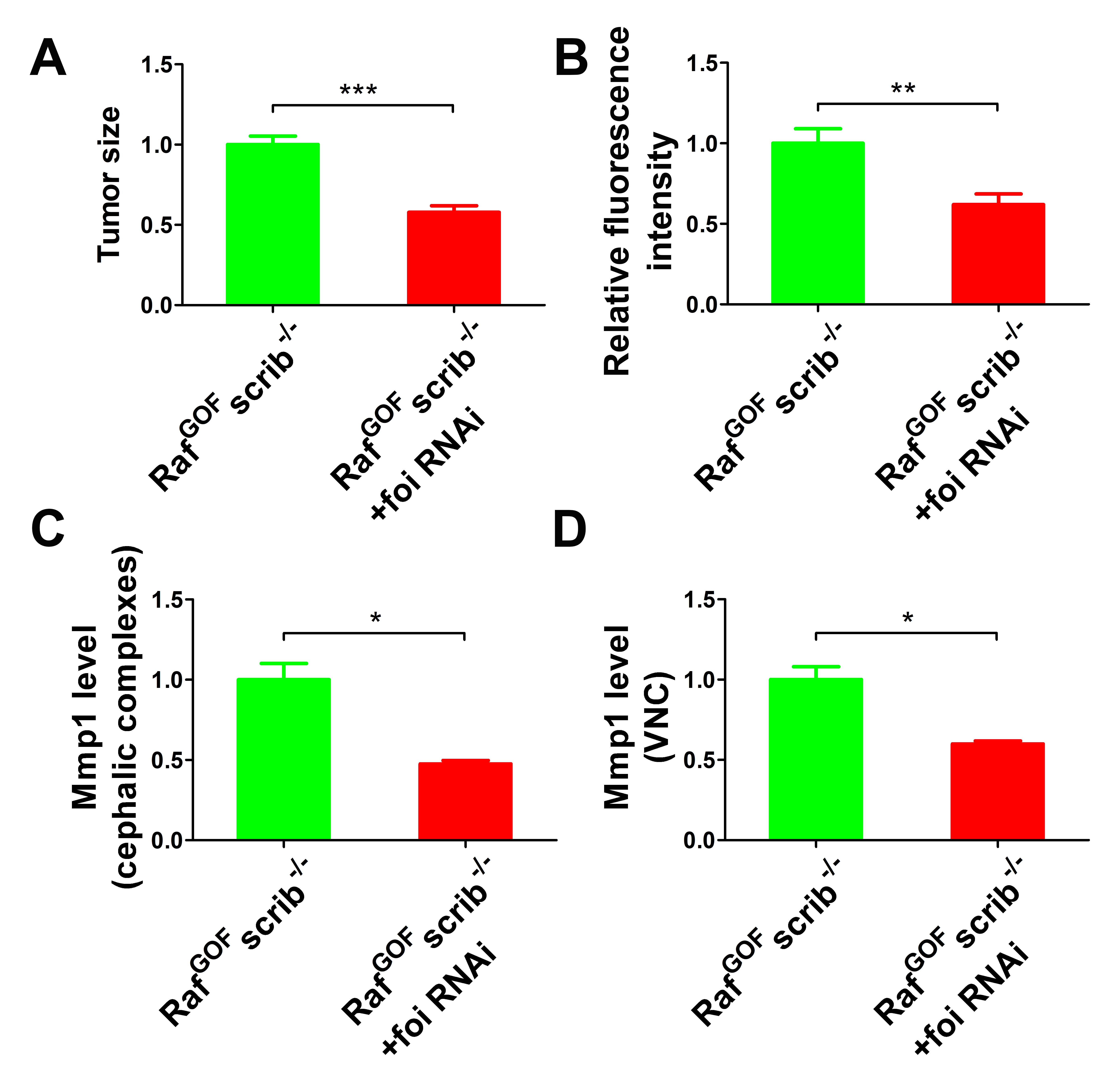


**Figure S5. *foi* RNAi inhibited tumour growth and invasion. (A-B)** Quantification of tumour size and fluorescence intensity in cephalic complex at 12 d AEL. n=16 (Raf^GOF^scrib^-/-^), n=18 (Raf^GOF^scrib^-/-^, *foi* RNAi). ** *P* < 0.01. **(C-D)** Quantification of Mmp1 level in cephalic complex and VNC at 12 d AEL. n=5. * *P* <0.05. Genotypes used are as follows: **(A-D)** *ey-Flp/+; Act-y^+^-Gal4, UAS-GFP/+; FRT82B, scrib^1^/FRT82B, UAS-Raf^GOF^, scrib^1^, ey-Flp/+; Act-y^+^-Gal4, UAS-GFP/foi RNAi; FRT82B, scrib^1^/FRT82B, UAS-Raf^GOF^, scrib^1^.*


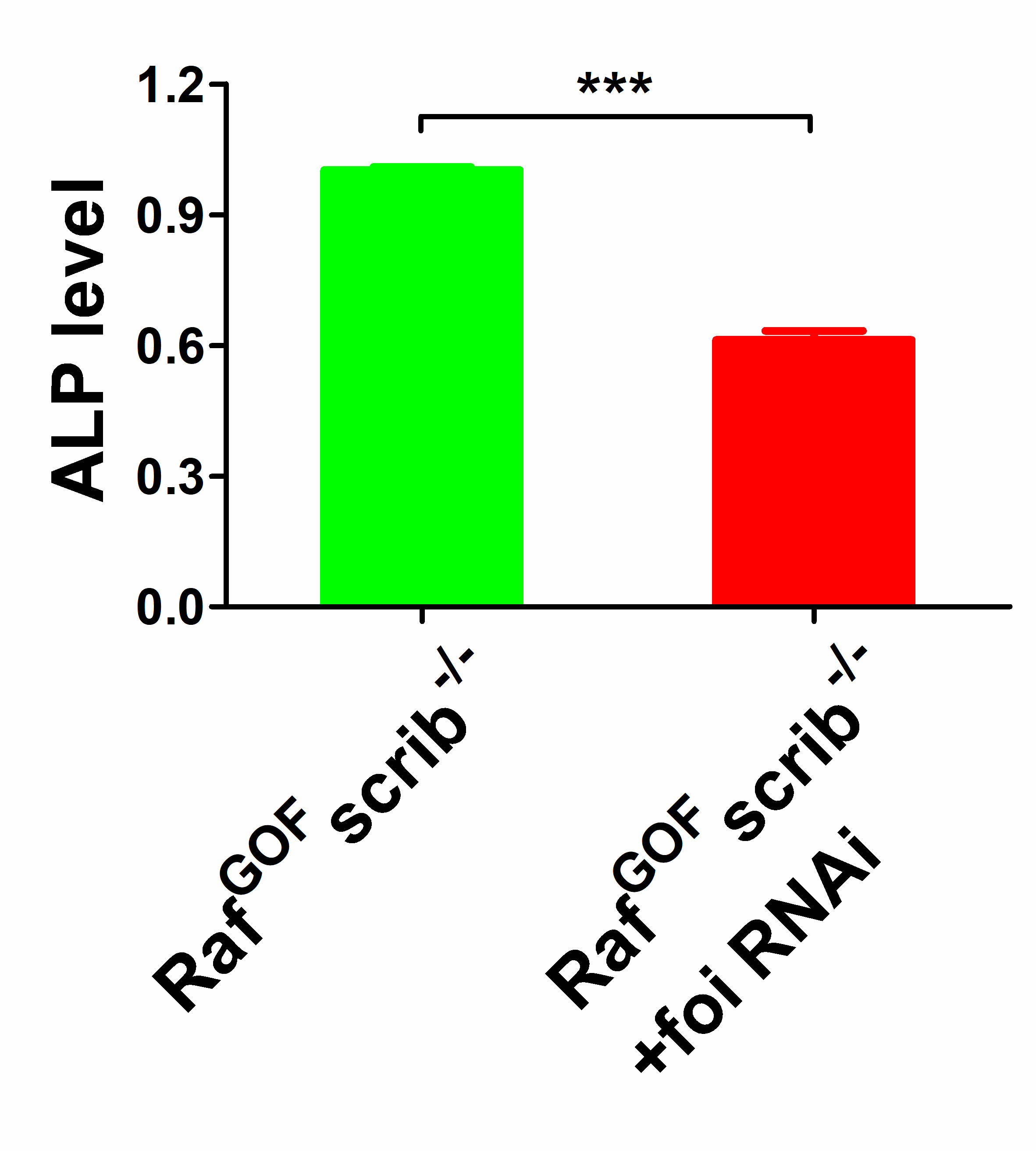


**Figure S6. *foi* RNAi** **resulted in decreased zinc level in the tumour.** The flies were crossed and reared on standard cornmeal media at 25℃, n=3. Genotypes used are as follows: *ey-Flp/+; Act-y^+^-Gal4, UAS-GFP/+; FRT82B, tub-GAL80/+, ey-Flp/+; Act-y^+^-Gal4, UAS-GFP/foi RNAi; FRT82B, scrib^1^/FRT82B, UAS-Raf^GOF^, scrib^1^*.
